# A Novel Nitronyl Nitroxide with Salicylic Acid Framework Attenuates Pain Hypersensitivity and Ectopic Neuronal Discharges in Radicular Low Back Pain

**DOI:** 10.1155/2015/752782

**Published:** 2015-11-01

**Authors:** Wen-Juan Han, Lei Chen, Hai-Bo Wang, Xiang-Zeng Liu, San-Jue Hu, Xiao-Li Sun, Ceng Luo

**Affiliations:** ^1^Institute of Neuroscience, Fourth Military Medical University, Xi'an 710032, China; ^2^Department of Pharmacy, Xijing Hospital, Fourth Military Medical University, Xi'an 710032, China; ^3^School of Pharmacy, Fourth Military Medical University, Xi'an 710032, China; ^4^Class 2013, School of Clinical Medicine, Fourth Military Medical University, Xi'an 710032, China

## Abstract

Evidence has accumulated that reactive oxygen species and inflammation play crucial roles in the development of chronic pain, including radicular low back pain. Nonsteroid anti-inflammatory drugs (NSAIDs), for example, salicylic acid, aspirin, provided analgesic effects in various types of pain. However, long-term use of these drugs causes unwanted side effects, which limits their implication. Stable nitronyl (NIT) nitroxide radicals have been extensively studied as a unique and interesting class of new antioxidants for protection against oxidative damage. The present study synthesized a novel NIT nitroxide radical with salicylic acid framework (SANR) to provide synergistic effect of both antioxidation and antiinflammation. We demonstrated for the first time that both acute and repeated SANR treatment exerted dramatic analgesic effect in radicular low back pain mimicked by chronic compression of dorsal root ganglion in rats. This analgesic potency was more potent than that produced by classical NSAIDs aspirin and traditional nitroxide radical Tempol alone. Furthermore, SANR-induced behavioral analgesia is found to be mediated, at least in partial, by a reduction of ectopic spontaneous discharges in injured DRG neurons. Therefore, the synthesized NIT nitroxide radical coupling with salicylic acid framework may represent a novel potential therapeutic candidate for treatment of chronic pain, including radicular low back pain.

## 1. Introduction

Radicular low back pain represents a frequent and poorly understood medical problem. It is a major cause of disability and higher health care costs in the world. This clinical condition often results from vertebral injuries, intervertebral disc herniation, intervertebral foramen stenosis, or other disorders affecting the dorsal root ganglion (DRG) or its near nerve root. So far, to reveal the underlying mechanism of radicular low back pain, a number of preclinical models have been developed that attempt to mimic the above known causes of low back pain [1, 2]. Amongst those, chronic compression of the dorsal root ganglion (CCD) model in rodents displayed dramatic pain hypersensitivity such as mechanical hypersensitivity (hyperalgesia and allodynia) and thermal hyperalgesia that mimic the pain symptom observed in low back pain patients [1–6]. Although epidural steroid injection and surgical intervention have been used both clinically and experimentally in many cases, radicular low back pain remains a common chronic pain condition that is sometimes refractory to current treatment modalities [7, 8]. Therefore, development of new therapeutics is helpful and in urgent need towards the treatment of radicular low back pain.

**Figure 1:**
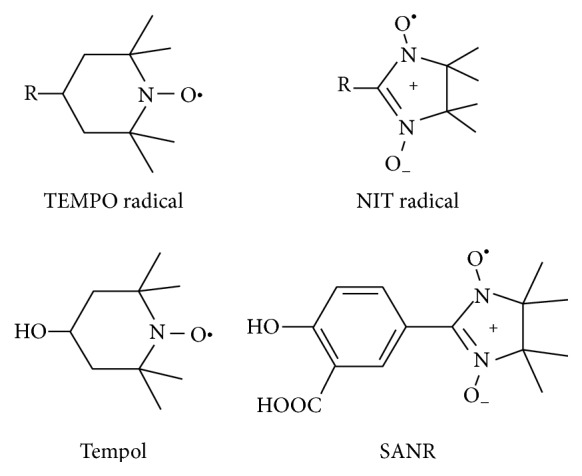
Structure of SANR and other nitroxide radicals. Structure of TEMPO, *α*-nitronyl (NIT) group radicals, Tempol, and SANR.

Much evidence has accumulated that reactive oxygen species (ROS) play an important role in the development of chronic pain [9, 10]. Various ROS scavengers and antioxidants provided analgesic effects in animal models of inflammatory and neuropathic pain [9, 11–15]. In recent years, nitroxide radicals have been extensively studied as a unique and interesting class of antioxidants to protect against ionizing radiation [16], ischemia/reperfusion injury [17], neurodegenerative diseases [18, 19], and chronic pain [9, 11–14]. Some nitroxide radicals, for example, amifostine, are being used in clinical practice [20]. Unlike other antioxidants that act in a sacrificial mode, nitroxide radicals act as self-replenishing antioxidants in a catalytic manner. The TEMPO and *α*-nitronyl (NIT) groups are the two major kinds of nitroxide radicals (Figure 1). Tempol (4-hydroxy-2,2,6,6-tetramethylpiper-idine-N-oxyl), a kind of TEMPO, is found to alleviate pain in various experimental pain models [12–15, 21]. Compared with Tempol, NIT group nitroxide radicals have an extensive distribution of unpaired spin density. As such, much attention has been levied towards the development of this kind of new NIT nitroxyl radicals. However, whether NIT group NRs possess antinociceptive action has remained elusive.

In addition to reactive oxygen species, inflammatory processes are thought to play key roles in radicular low back pain (please see Strong et al. [1] for review). Attenuation of pain behaviors by nonsteroidal anti-inflammatory drugs (NSAIDs) has been demonstrated in various rodent back pain models [22–26]. However, long-term use of NSAIDs causes unwanted gastrointestinal and cardiovascular side effects, which limits its broader applications. Herein, we have for the first time synthesized a novel NIT nitroxide radical with salicylic acid framework (SANR) exerting the beneficial effects of both the NSAIDs and the antioxidants (Figure 1). We demonstrated that both acute and long-term systemic administration of SANR attenuated the mechanical hypersensitivity and thermal hyperalgesia observed in an experimental model of CCD rats. Furthermore, SANR provided more pain relief than either traditional nitroxide compound Tempol or NSAIDs aspirin alone.

Evidence has accumulated that abnormal spontaneous discharges of primary afferent sensory neurons may contribute to radicular low back pain [3, 4]. We therefore investigated whether SANR produced analgesia by inhibition of spontaneous discharges of primary afferent sensory neurons in CCD rats. Our results revealed that SANR produced a marked depression of spontaneous discharges of primary afferent A fibers in CCD rats in a dose-dependent manner. When compared to the same concentration of Tempol or aspirin, SANR exhibited a much stronger analgesic effect. Taken together, these findings clearly suggest that synthesis of new NIT nitroxyl radical with salicylic acid framework may represent a potential new candidate for the treatment of radicular low back pain.

## 2. Materials and Methods

### 2.1. Synthesis of SANR

2,3-Bis(hydroxyl amino)-2,3-dimethylbutane was prepared by the published method. All other chemical reagents were purchased from the Beijing OuHe Chemical Limited Company (Beijing, China). The other chemical reagents were used without further purification. High resolution mass spectroscopy (HRMS) was carried out on a Varian 7.0T ESI-FTICR-MS (Varian, USA). Elemental analyses were carried out using a PerkinElmer analyser model 240C (PerkinElmer, America).

A solution of 5-formyl-2-hydroxybenzoic acid (1.66 g, 10 mmol) and 2,3-bi (hydroxyl amino)-2,3-dimethylbutane (1.48 g, 10 mmol) in methanol (25 mL) was heated under reflux for 18 h. After the reaction, the methanol was removed and the residue was suspended in 100 mL CH_2_Cl_2_, aqueous NaIO_4_ (2.14 g, 10 mmol in 50 mL) was added dropwise over a period of 10 min at 0°C, and the mixture was stirred for a further 20 min at 0°C. The organic phase was separated, and the aqueous phase was extracted with CH_2_Cl_2_ (3 × 20 mL). The combined organic layers were dried over anhydrous Na_2_SO_4_. The deep blue solution was then evaporated. The crude product was purified by column chromatography on silica gel using absolute ether/ethyl acetate/(1 : 1) as eluent, giving a deep blue solid product 1. Yield 21.4%. MS (*m/z*): 294.10 [M+H]^+^; Anal. Calcd for C_14_H_17_N_2_O_5_: C, 57.33; H, 5.84; N, 9.55%, Found: C, 57.35; H, 5.81; N, 9.67%.

### 2.2. Animals and CCD Models

Adult Sprague-Dawley rats weighing 200–250 g were subjected to CCD surgery. All experimental protocols were approved by the Institutional Animal Use and Protection Committee, Fourth Military Medical University. All the testing was carried out in accordance with the approved guidelines. The CCD model was prepared as described previously [3]. Briefly, under anesthesia (sodium pentobarbital, 40 mg/kg, i.p.), the transverse process and intervertebral foramen at L5 on left side were exposed. A L-shaped stainless steel rod was inserted into foramen to produce a steady compression on the ganglia. After the rods were in place, the muscle and skin layers were sutured with administration of about 200 mg antibiotics.

### 2.3. Behavioral Tests

Behavioral testing was carried out in habituated mice by an observer blinded to the identity of the groups. As previously described [27, 28], mechanical sensitivity was tested with manual application of von Frey hairs (North Coast) to the plantar surface of hindpaw. Each filament was applied 10 times and the paw withdrawal response frequency (the percentage of positive responses to the stimulus) was recorded. The force of a particular filament required to elicit 50% frequency of paw withdrawal was expressed as the mechanical threshold. Thermal sensitivity was tested by application of infrared heat to the plantar surface of hindpaw and the response latency was measured from an automated device readout (IITC Life Science).

Mice were evaluated for their motor coordination using the rotarod test. After training sessions, mice were placed on an accelerating rod. The latency for the rat to fall off the rod and the speed of rod at this time were recorded and a mean latency and speed for the 4 trials were calculated. The acceleration was from 2 to 60 r.p.m. over a 180 s period.

### 2.4. Extracellular Recording of DRG Single Unit Fiber Activities

Activities of single unit DRG A-fibers were recorded 3–8 days after the CCD surgery. Under sodium pentobarbital anesthesia (40 mg/kg, i.p.), laminectomy was performed at the L1-L2 and L4-L5 levels, and two small pools were formed at the exposure regions, separately. In the L4-L5 pool the stainless steel rod was removed. The spinal nerve was transected 7–10 mm distal to the DRG so that the discharge activities of the dorsal root fibers would originate primarily from the DRG region and not from peripheral sensory terminals. During recording, L4-L5 pool (drug pool) was filled with warm ACSF (35–37°C) containing (in mM): NaCl 150, KCl 5, CaCl_2_ 2, MgCl_2_ 1, D-glucose 10, and HEPES 10, with the pH adjusted to 7.4, and L1-L2 pool (recording pool) was filled with warm paraffin oil (35–37°C). Under a microscope, a microfilament (15–30 *μ*m in diameter) presumably including up to a few nerve fibers was teased from the dorsal root. The proximal end was placed on a fine platinum electrode (29 *μ*m in diameter) for electrophysiological recording of DRG single fiber activities. The firing patterns of a single fiber were displayed on a memory oscilloscope (VC-11, Japan) and recorded via an A/D board to a computer hard drive and stored for offline analysis. Unit activities with identical wave forms were selected as single fiber activities as previously described [3, 29].

Aspirin was dissolved in dimethyl sulfoxide (DMSO) as a stock solution and kept frozen; it was diluted in ACSF before the experiments. Tempol and SANR were dissolved in ACSF, respectively, before the experiments. Single unit discharges were recorded in the presence and absence of these three drugs or vehicle for at least another 5 min. Inhibition rate in discharge were calculated as (maximal discharge rate after drug using − baseline rate)/baseline rate × 100% [30].

### 2.5. Statistical Analysis

All data are expressed as mean ± S.E.M. Analysis of variance (ANOVA) for random measures was carried out, followed by either a post hoc Fisher's test or Dunnett's test. *P* < 0.05 was considered significant.

## 3. Results

### 3.1. Synthesis of SANR

**Figure 2:**
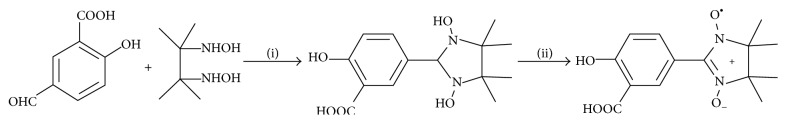
Synthesis of SANR. A scheme showing the synthetic route of SANR. Reagents and conditions: (i) MeOH, r.t.; (ii) NaIO_4_, CH_2_Cl_2_, 0°C.

The compound SANR was synthesized according to Ullman's procedure as shown in Figure 2. According to Ullman's pioneering work, any aldehydes may give rise to NIT nitroxides [31]. Followed by condensation of 5-formyl-2-hydroxybenzoic acid with 2,3-bis(hydroxyl amino)-2,3-dimethyl butane in methanol solution at room temperature, stable white solids 1,3-dihydroxyimidazolidine were rapidly obtained [32]. One of the key steps in the synthesis of NIT nitroxide radicals is the oxidation of 1,3-dihydroxyimidazolidines. We chose the aqueous of NaIO_4_ as oxidant to oxidize the 1,3-dihydroxyimidazolidine to obtain the final target compound SANR in yield of 21%.

### 3.2. Development of Mechanical Hypersensitivity and Thermal Hyperalgesia in Rats Subjected to Chronic Compression of DRG (CCD)

**Figure 3:**
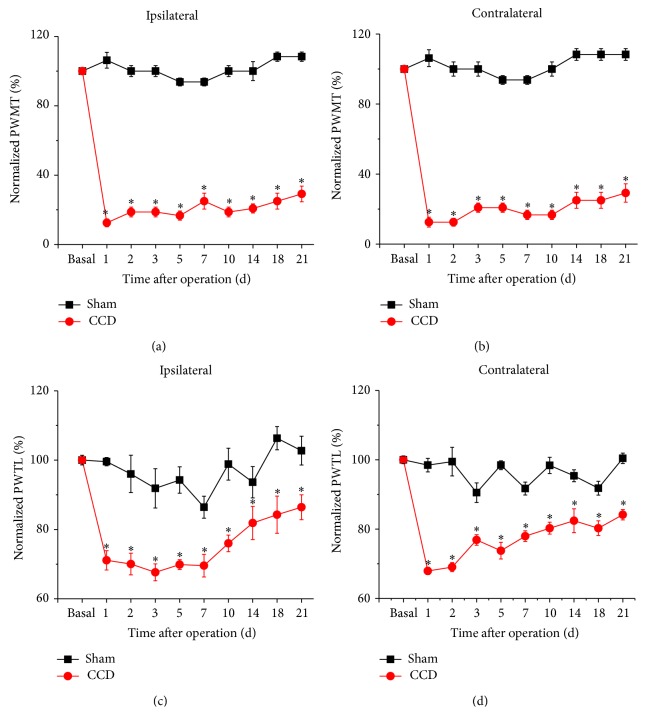
Development of mechanical hypersensitivity and thermal hyperalgesia following chronic compression of DRG (CCD) in rats. (a-b) Mechanical hypersensitivity developed in CCD rats but not in sham controls for both ipsilateral (a) and contralateral hindpaws (b). Note that paw withdrawal mechanical threshold (PWMT) to von Frey hairs decreased dramatically from the 1st day following chronic compression of L5 DRG (*n* = 10; *P* < 0.05). (c-d) Thermal hyperalgesia was observed by measuring paw withdrawal thermal latency (PWTL) to radiant heat in CCD rats and sham controls for both ipsilateral (c) and contralateral hindpaws (d). Note that PWTL significantly decreased in CCD rats (*n* = 10; *P* < 0.05) but not in sham group (*n* = 10; *P* > 0.05) with a similar time course with mechanical hypersensitivity. All data are expressed as mean ± S.E.M.

Following chronic compression of L5 DRG (CCD), the rats appeared in good health and did not show any signs of autotomy throughout the study. Sensitivity of CCD rats to mechanical and thermal stimuli was tested at different time points after operation. Compared to sham controls, CCD rats developed bilateral mechanical hypersensitivity (allodynia and hyperalgesia), which was manifested as a significant decrease in response threshold to von Frey hairs application to the bilateral hindpaws (Figures 3(a) and 3(b), *n* = 10, *P* < 0.05 at all time points). This mechanical hypersensitivity appeared on the 1st day after compression, persisting over the entire experimental period. In parallel, a dramatic drop in response latency to noxious plantar heat stimuli, reflecting thermal hyperalgesia, was found in bilateral hindpaws of CCD rats (Figures 3(c) and 3(d), *n* = 10, *P* < 0.05 at all time points). Therefore, it can be inferred that rats with chronic compression of L5 DRGs develop strong mechanical hypersensitivity and thermal hyperalgesia, which is consistent with previous reports in rodents [3–5].

### 3.3. Acute Administration of SANR Attenuates Mechanical Hypersensitivity and Thermal Hyperalgesia in CCD Rats

**Figure 4:**
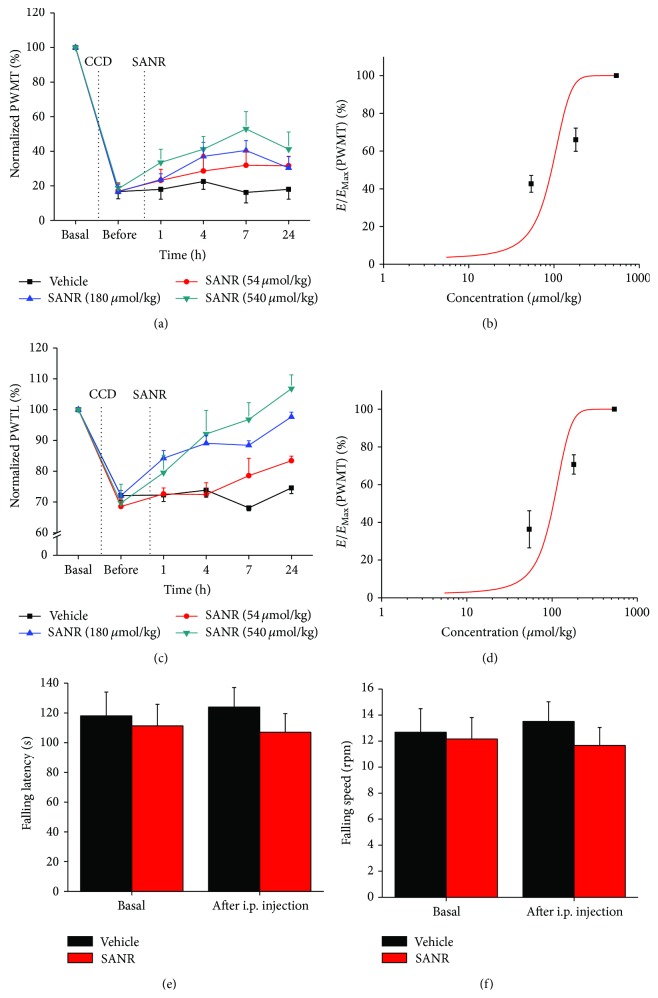
(a, b) Intraperitoneal (i.p.) administration of SANR (54, 180, and 540 *μ*mol/kg body weight) attenuated mechanical hypersensitivity (a) and thermal hyperalgesia (c) induced by chronic compression of lumbar DRG in a dose-dependent manner (*n* = 10). Concentration-response curves of SANR at 7 h after drug delivery on mechanical hypersensitivity (b) and thermal hyperalgesia (d) are shown, respectively. (e, f) Motor coordination was not altered by systemic SANR (180 *μ*mol/kg, i.p.). Quantitative analysis showing that i.p. SANR did not affect the latency (e) and speed (f) for rats falling from the accelerating rod, as compared to vehicle group (*n* = 6 for each group, *P* > 0.05). All data are expressed as mean ± S.E.M.

To investigate the acute effect of SANR on the pain hypersensitivity observed in CCD rats, we administered SANR via intraperitoneal (i.p.) injection once at 3 d after operation when mechanical and thermal hyperalgesia was completely developed. As shown in Figure 4(a), acute administration of SANR (54, 180, 540 *μ*mol/kg body weight) dose-dependently elevated the mechanical threshold to von Frey hairs in ipsilateral paw as compared to predrug level, reflecting as attenuation of mechanical hypersensitivity (Figure 4(a), *n* = 10). This antinociceptive effect started from 1 h after drug administration, persisting over the test period, namely, 24 h after SANR delivery. Dose-response curve at 7 h after drug was fitted to a Hill equation, which yielded an IC50 of 506.5 ± 14.6 *μ*mol/kg for SANR in attenuation of mechanical hypersensitivity (Figure 4(b)). Similarly, CCD-induced ipsilateral thermal hyperalgesia was dramatically reduced by acute SANR in a dose-dependent manner with a similar time course, as measured by a prolongation of response latency to radiant heat stimuli compared to predrug level (Figure 4(c), *n* = 10). The dose-response curve revealed an IC50 of 119.9 ± 4.1 *μ*mol/kg for SANR in inhibition of thermal hyperalgesia (Figure 4(d)). In contrast, i.p. vehicle did not alter the magnitude of CCD-induced mechanical hypersensitivity and thermal hyperalgesia (Figures 4(a) and 4(c), *n* = 10, *P* > 0.05). In addition, motor coordination was not altered by systemic SANR administration (180 *μ*mol/kg, i.p.), as compared to vehicle group (Figures 4(e) and 4(f), *P* > 0.05, *n* = 6).

**Figure 5:**
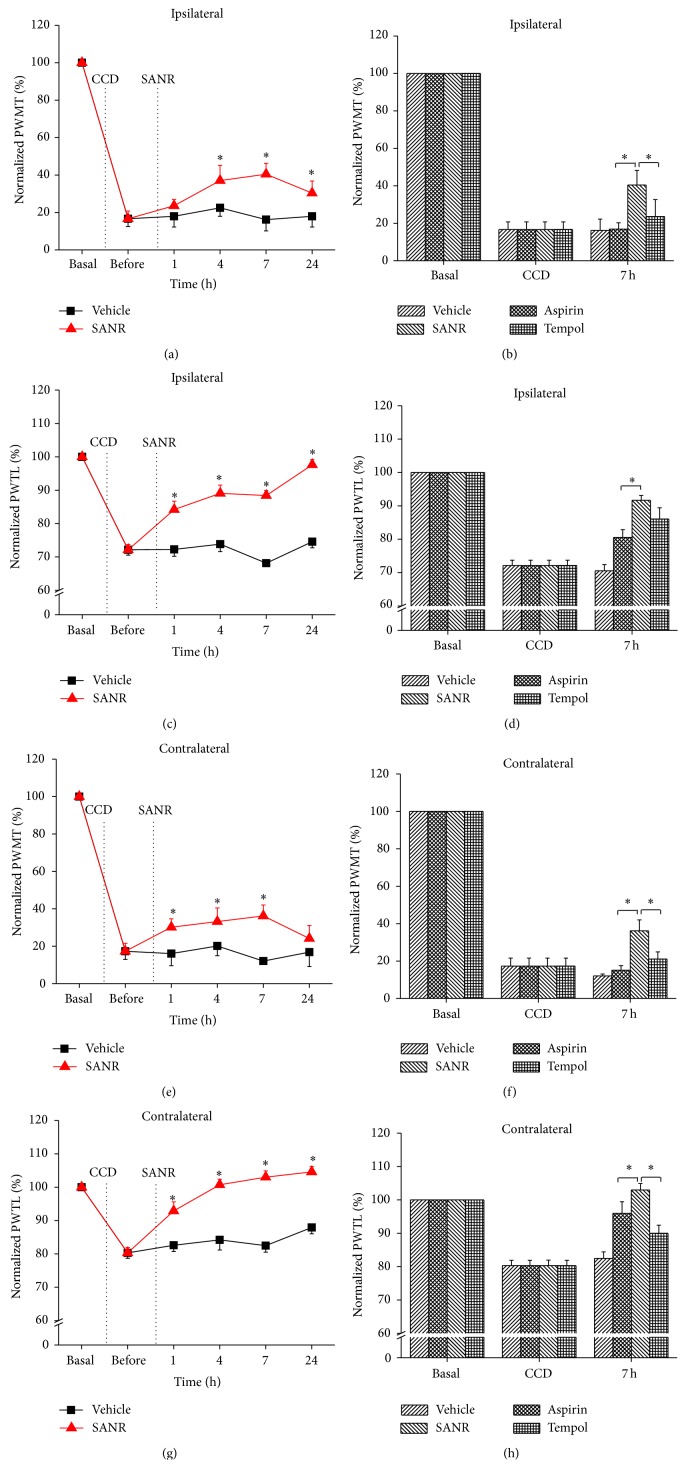
Acute treatment of SANR produces antinociceptive effect on CCD-induced mechanical hypersensitivity and thermal hyperalgesia in bilateral hindpaws, which is more potent than parent nitroxide moiety Tempol and parent NSAIDs moiety aspirin. (a, c) Time course of mechanical hypersensitivity (a) and thermal hyperalgesia (c) in ipsilateral hindpaw of CCD rats before and after i.p. SANR (180 *μ*mol/kg) (red) and vehicle treatment (black). Note that ipsilateral mechanical and thermal hyperalgesia was attenuated significantly by SANR. (b, d) Comparison of analgesic potency in ipsilateral mechanical (b) and thermal hyperalgesia (d) produced by the same concentration of SANR, Tempol, and aspirin. Note that SANR showed significantly stronger effect than Tempol and aspirin. (e, g) Contralateral mechanical hypersensitivity (e) and thermal hyperalgesia (g) was reduced by acute SANR as well. (f, h) Comparison of the efficacy by SANR with Tempol and aspirin in inhibiting mechanical (f) and thermal hyperalgesia (h). All data are expressed as mean ± S.E.M. ^∗^ *P* < 0.05.

We then further compared the analgesic potency of SANR with the same dosing regimen of traditional nitroxide compound Tempol and classical NSAIDs aspirin alone. As shown in Figure 5(a), SANR at dose of 180 *μ*mol/kg significantly inhibited ipsilateral mechanical hypersensitivity. In contrast, i.p. Tempol and aspirin alone at this low concentration produced little effect on the mechanical hypersensitivity. Quantitative analysis at 7 h after drug delivery revealed that the analgesic potency by SANR was significantly higher than Tempol and aspirin alone (Figure 5(b), *P* = 0.045 versus Tempol, *P* = 0.006 versus aspirin). In parallel, SANR exerted stronger inhibition on ipsilateral thermal hyperalgesia when compared to Tempol and aspirin alone (Figures 5(c) and 5(d), *n* = 10, *P* = 0.047 versus Tempol, *P* = 0.025 versus aspirin).

In addition to prominent inhibition on ipsilateral pain hypersensitivity, acute SANR produced marked reduction of contralateral spread of mechanical hypersensitivity and thermal hyperalgesia as well (Figures 5(e) and 5(g), *n* = 10, *P* < 0.05). Similarly, SANR was found to be more efficacious on depression of both contralateral mechanical (Figure 5(f), *P* = 0.044 versus Tempol, *P* = 0.032 versus aspirin) and thermal hyperalgesia than Tempol and aspirin alone (Figure 5(h), *P* = 0.018 versus Tempol, *P* = 0.046 versus aspirin).

### 3.4. Repeated SANR Treatment Progressively Reverses the Development of CCD-Induced Mechanical Hypersensitivity and Thermal Hyperalgesia

**Figure 6:**
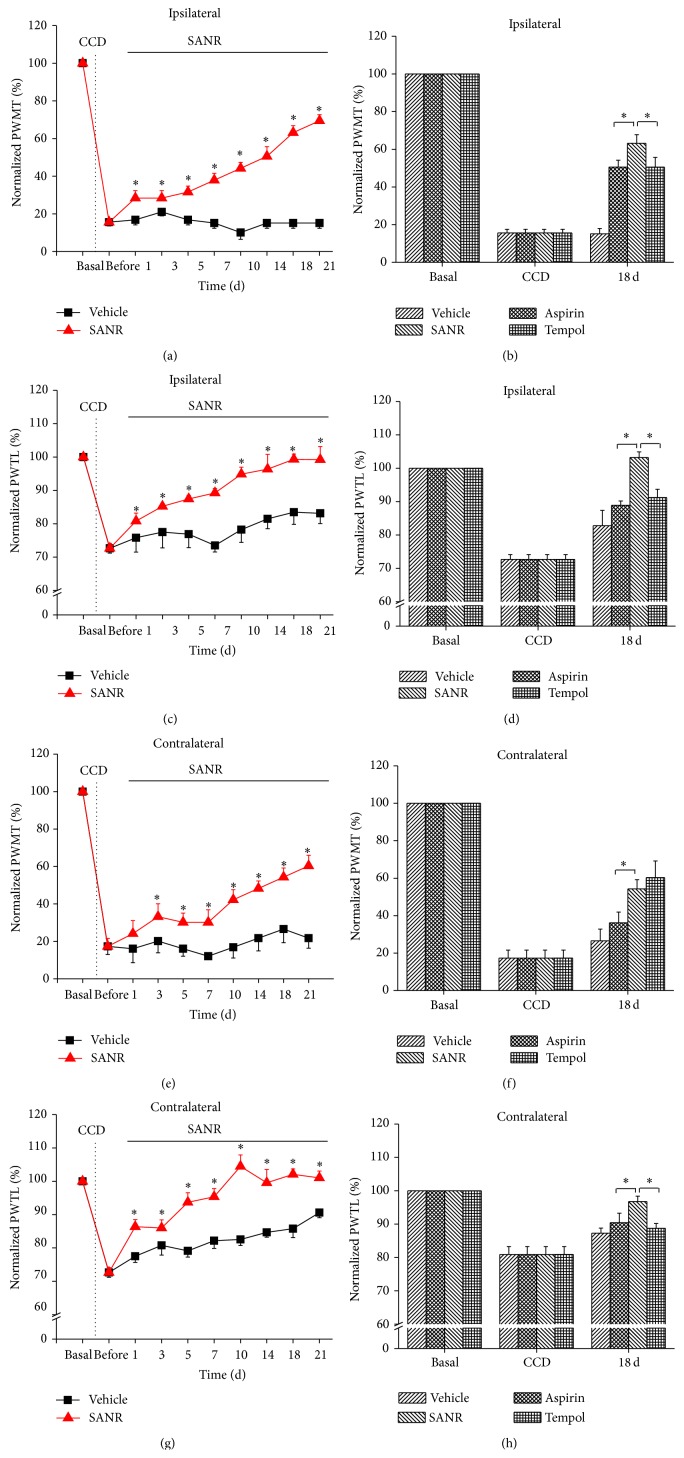
Repeated treatment of SANR produces antinociceptive effect on CCD-induced mechanical hypersensitivity and thermal hyperalgesia in bilateral hindpaws, which is more potent than parent nitroxide moiety Tempol and parent NSAIDs moiety aspirin. (a, c) Time course of mechanical hypersensitivity (a) and thermal hyperalgesia (c) in ipsilateral hindpaw of CCD rats before and after repeated i.p. SANR (180 *μ*mol/kg) (red) and vehicle treatment (black). Note that ipsilateral mechanical and thermal hyperalgesia was progressively and completely reversed by SANR. (b, d) Comparison of analgesic potency in ipsilateral mechanical (b) and thermal hyperalgesia (d) produced by the same concentration of SANR, Tempol, and aspirin for 18 days. Note that SANR showed significantly stronger effect than Tempol and aspirin. (e, g) Contralateral mechanical hypersensitivity (e) and thermal hyperalgesia (g) were reduced by repeated SANR administration as well. (f, h) Comparison of the efficacy by SANR with Tempol and aspirin in inhibiting mechanical (f) and thermal hyperalgesia (h) at 18 d during continuous drug administration. All data are expressed as mean ± S.E.M. ^∗^ *P* < 0.05.

We further addressed whether repeated treatment of SANR could reverse the development of CCD-induced pain hypersensitivity. To do this, we administered SANR for 21 d once daily beginning at 3 d after DRG compression. As compared to vehicle group, repeated administration of SANR progressively reversed the development of ipsilateral mechanical hypersensitivity in CCD rats (Figure 6(a), *n* = 10, *P* < 0.05 at all time points tested). In comparison with Tempol and aspirin, SANR produced more pain relief on mechanical hyperalgesia (Figure 6(b), *n* = 10 for each drug). Quantitative analysis at 18 d after drug treatment showed that the extent of reversal of mechanical hyperalgesia by SANR was significantly stronger than that of Tempol and aspirin (Figure 6(b), *P* = 0.043 versus Tempol, *P* = 0.039 versus aspirin). Similarly, progressive and complete reversal of ipsilateral thermal hyperalgesia was seen after treatment with repeated SANR (Figure 6(c), *n* = 10). Furthermore, SANR was much more efficacious than Tempol and aspirin alone (Figure 6(d), *P* = 0.029 versus Tempol, *P* = 0.018 versus aspirin).

Figures 6(e) and 6(g) show a notable depression of contralateral mechanical hypersensitivity (Figure 6(e), *n* = 10) and thermal hyperalgesia by SANR (Figure 6(g), *n* = 10). Although Tempol displayed a comparable effect on contralateral mechanical hypersensitivity with SANR when examined at 18 d after drug application (Figure 6(f), *n* = 10, *P* = 0.059), its inhibition on thermal hyperalgesia was still weaker than SANR (Figure 6(h), *n* = 10, *P* = 0.036). Aspirin was consistently weaker than SANR on attenuation of both contralateral mechanical hypersensitivity (Figure 6(f), *n* = 10, *P* = 0.047) and thermal hyperalgesia (Figure 6(h), *n* = 10, *P* = 0.049). Collectively, these behavioral results uncovered a potential therapeutic value of this newly synthesized compound SANR on the development of hyperalgesia observed in radicular low back pain.

### 3.5. SANR Inhibits Ectopic Spontaneous Discharges in Compressed DRG Neurons

**Figure 7:**
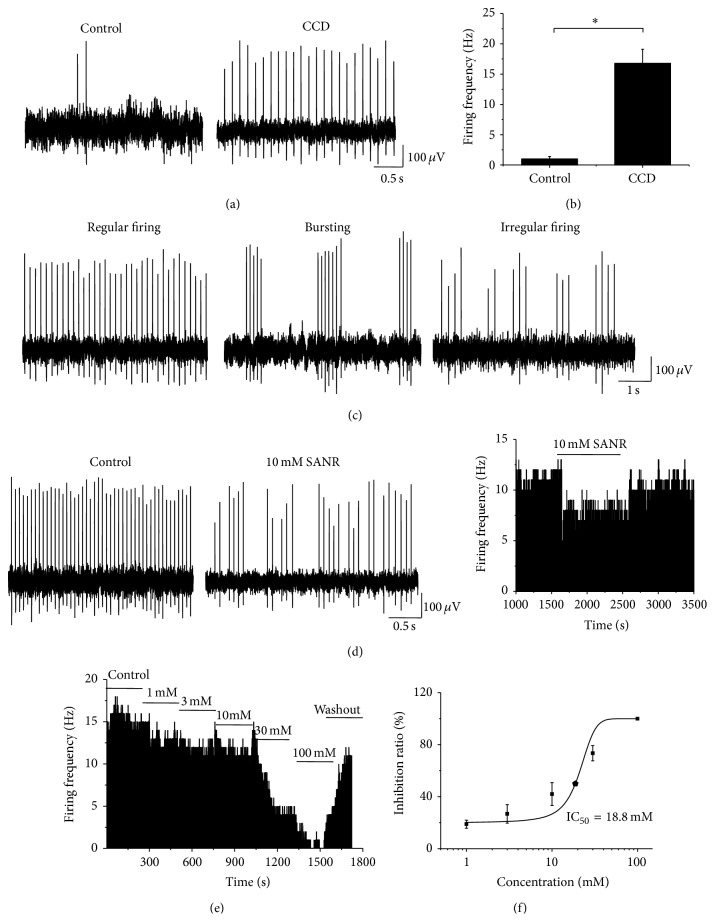
SANR produces dramatic inhibition on ectopic spontaneous discharges of the injured DRG neurons in CCD rats. (a) Following chronic compression of DRG, primary afferent A fibers of DRG neurons frequently exhibited ectopic spontaneous discharges (right panel); this rarely happens in sham control rats (left panel). (b) Quantitative analysis of the rate of spontaneous discharges in A-type DRG neurons derived from CCD and control rats. (c) According to the dynamic features of interspike interval series, three different firing patterns were observed, which are regular (left panel), bursting (middle panel), and irregular patterns (right panel). (d) Representative traces showing that bath application of SANR (10 mM) (middle panel) produces marked depression of spontaneous discharge rate compared to vehicle in the same cell (left panel). Frequency histogram on the same cell was shown in the right panel. (e) Frequency histogram showing a dose-dependent inhibition of ectopic spontaneous discharges by SANR in a reversible manner. (f) Analysis of the curve based on the Hill plot yielded an IC_50_ of 18.8 mM for SANR.

Previous studies have shown that abnormal spontaneous discharges of primary afferent sensory neurons may contribute to the development of radicular low back pain [3, 4]. We are therefore interested to know whether SANR-induced analgesia in low back pain was mediated by its suppression on ectopic spontaneous discharges in compressed DRG neurons. Single fiber recordings were employed in the dorsal roots of the injured L5 DRG derived from 27 CCD rats. Upon testing at 3–5 d after the onset of compression, we observed that primary afferent A fibers frequently exhibited ectopic spontaneous discharges (Figure 7(a), right panel). The conduction velocities were within the range of 2.5–26 m/s indicating that all the axons were myelinated. This ectopic spontaneous discharge rarely happens in control rats (Figure 7(a), left panel). Quantitative analysis showed that the mean frequency of spontaneous firing reached 16.78 ± 2.33 Hz (Figure 7(b), *n* = 118). This spontaneous activity of all the tested units can last for 2–6 h (2.28 ± 1.35 h). According to the dynamic features of interspike interval series, three different firing patterns have been previously reported in the injured lumbar A-fiber DRG neurons, which is periodic, nonperiodic (irregular), and bursting activity [3]. In our case, most patterns of spontaneous firing (59/118) observed fell into regular discharges (Figure 7(c), left panel). 37/118 were bursting activity (Figure 7(c), middle panel), and 22 out of 118 fibers displayed irregular firings (Figure 7(c), right panel).

As compared to control, superfusing SANR at a concentration of 10 mM reversibly inhibited the rate of spontaneous discharges of the primary afferent A fibers from CCD rats (see Figure 7(d) left and middle panels for original traces and Figure 7(d) right panel for frequency histogram of typical examples). When examined in a concentration range of 1–100 mM, the inhibitory rate of SANR was enhanced in magnitude with increasing concentrations, as seen in Figure 7(e).  Figure 7(f) illustrates a dose-response curve for the suppressive effect of SANR on spontaneous activity (Figure 7(f), *n* = 7). The inhibitory rate averaged to be 19.4 ± 5.5%, 31.2 ± 11.6%, 43.7 ± 15.8%, 80.6 ± 5.4%, and 98.4 ± 1.5% for SANR at 1, 3, 10, 30, and 100 mM, respectively. Analysis of the curve based on the Hill plot yielded an IC_50_ of 18.8 mM for SANR (Figure 7(f)). These results indicate that SANR-induced analgesia may be at least, partially mediated by its inhibition of ectopic spontaneous activity of injured DRG neurons.

### 3.6. SANR Exerts Stronger Inhibition of Spontaneous Activity of Injured DRG Neurons Than Tempol and Aspirin

**Figure 8:**
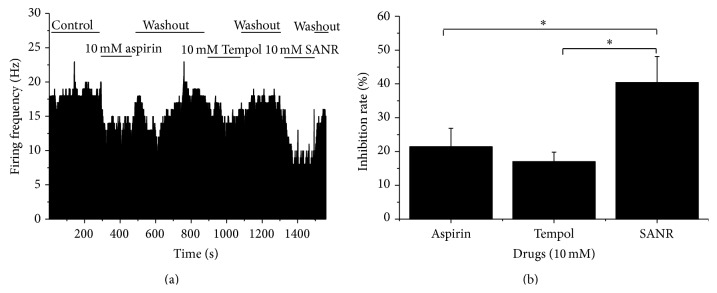
Comparison of the inhibitory potency of SANR (10 mM) on ectopic spontaneous discharge with the same concentration of Tempol and aspirin. (a) Frequency histogram showing the inhibition of ectopic spontaneous discharge of the injured DRG neurons by SANR, Tempol, and aspirin on the same cell. (b) Quantitative analysis showing that the inhibition rate by SANR (*n* = 4) on spontaneous activity was much stronger than that by Tempol (*n* = 4, *P* < 0.05) and aspirin (*n* = 4, *P* < 0.05) alone. All data are expressed as mean ± S.E.M.

We further compared the efficacy of SANR with the same concentration of Tempol and aspirin alone on the ectopic spontaneous discharges in the injured DRG neurons. Three compounds were tested in the same cell. To exclude the possible influence between each other, the drug was applied in a mixed order. As shown in Figure 8(a), bath-applied aspirin at 10 mM significantly depressed the frequency of ectopic spontaneous discharges compared to control in a reversible manner (Figure 8(a), *n* = 4, *P* = 0.014). A similar effect was obtained upon Tempol (10 mM) treatment (Figure 8(a), *n* = 4, *P* = 0.019). In striking contrast, administration of SANR (10 mM) produced most potent inhibition of spontaneous activity (Figure 8(a), *n* = 4, *P* = 0.013). Quantitative analysis revealed that the averaged inhibition rate by SANR was significantly different from that by Tempol and aspirin alone (Figure 8(b), *n* = 4, *P* = 0.049 versus Tempol, *P* = 0.047 versus aspirin).

## 4. Discussion

Oxidative stress and inflammation have long been assumed to be involved in the development and maintenance of chronic pain [1, 9, 10]. Various antioxidants, for example, nitroxides and NSAIDs, for example, aspirin, have been shown to provide partial relief in some types of chronic pain [9, 11–15, 22–25]. However, chronic use of these drugs at high doses often causes severe unwanted side effects, which limits their broader implication. Utilizing chemical synthesis, the present study for the first time synthesized a new NIT nitroxide radical with salicylic acid framework (SANR).* In vivo* and* in vitro* results revealed that this new compound produced a remarkable antinociceptive effect in CCD-induced low back pain, which is much more potent than traditional nitroxide compound Tempol and classical NSAIDs aspirin. These results indicate that NIT nitroxide radical with salicylic acid framework might provide a new class of analgesic drug candidates.

A growing body of evidence has accumulated that stable nitroxide radicals have been studied as a unique and interesting class of antioxidants to protect again various oxidant stress including chronic pain [12–19, 21]. Stable nitroxides include two types: Tempol and NIT nitroxides. Although Tempol has been shown to exert antinociceptive effect in inflammatory and neuropathic pain [12–15, 21], whether NIT radicals protect against pain has not been fully understood. Compared with other antioxidants, NIT group nitroxides have incomparable advantages of scavenging radicals through a rapid catalytic manner. More importantly, electrochemical properties with more extensive distribution of the unpaired spin density make NIT group nitroxides more suitable for structure modification. For example, with the introduction of chirality into NIT nitroxides, a series of chiral NIT nitroxides were synthesized. These new compounds have been shown to be able to protect against radiation [33] and memory deficit [19]. These results inferred that modification of NIT nitroxides may represent a direction for the development of new therapeutic drugs against neurological disorders. However, whether NIT nitroxides and its derivatives possess analgesic effect has remained elusive. In this study, we successfully synthesized a new NIT nitroxide with salicylic acid framework with both antioxidative and anti-inflammatory action. In support of our assumption, Tempo-aspirin and Tempo-indomethacin have been recently synthesized and are found to exert anti-inflammatory and superoxide dismutase scavenging properties in A459 cells [34].

One of the most striking findings of this study is that SANR eliminated pain hypersensitivity in low back pain produced in an experimental of CCD model. Consistent with previous reports, CCD rats display not only ipsilateral but also contralateral mechanical and thermal hyperalgesia, indicating the involvement of peripheral and central sensitization [5, 35, 36]. Acute treatment with systemic SANR produced dramatic analgesic effect on bilateral mechanical hypersensitivity and thermal hyperalgesia lasting beyond 24 h. Following repeated administration of SANR, the development of bilateral pain hypersensitivity was progressively and completely reversed. This indicates that SANR may play a role in both peripheral and central sensitization induced by chronic compression. Furthermore, we compared the antihyperalgesic potency exerted by SANR with the same concentration of traditional nitroxides Tempol and classical NSAIDs aspirin alone. It was found that both acute and repeated treatment with SANR exhibited much stronger efficacy than Tempol and aspirin in expediting the recovery of low back pain. This suggests that the addition of NIT nitroxides moiety into NSAIDs salicylic acid framework may provide the additive analgesia via synergistic anti-inflammatory and antioxidative action.

Ectopic spontaneous activity of injured DRG neurons has been implicated as a key driver of neuropathic pain including radicular low back pain [3, 4, 37]. Previous reports together with our unpublished data showed that chronic neuropathic pain is often associated with spontaneous activity in A-type DRG neurons, whereas chronic inflammatory pain mainly induces spontaneous discharges in C-type DRG neurons [3, 4, 37, 38]. Consistent with previous studies, we demonstrated that A-type DRG neurons displayed frequent spontaneous firing following chronic DRG compression [3, 4, 30]. Another striking finding of the present study is that bath applications of SANR remarkably depressed the spontaneous discharges of A-type DRG neurons caused by DRG compression in a dose-dependent manner. This inhibition can be reversed after washout of SANR. Although NSAIDs aspirin and nitroxides Tempol have been reported to exert analgesic effect in some types of neuropathic pain [9, 11, 12, 22–25], the cellular mechanisms underlying this analgesia in CCD-induced low back pain has not been studied in details. This study showed that Tempol and aspirin exhibited significant inhibition on the spontaneous firing in injured A-type DRG neurons from CCD rats as well. However, when evaluating the inhibitory rate of the same concentration of these three compounds, SANR provided the strongest analgesia, which is significant from Tempol and aspirin. It can be inferred from the above that SANR-induced analgesia in low back pain* in vivo* may be mediated, at least in partial by the depression of ectopic spontaneous activity of injured DRG neurons.

The primary mechanisms of SANR underlying the behavioral and neuronal antinociceptive actions* in vivo* and* in vitro* have remained unclear. Several mechanisms have been implicated in the antinociceptive actions of aspirin [26], including inhibition of cyclooxygenases [39, 40], NF-*κ*B pathway [41], and acid-sensing ion channels [42] as well as activation of adenosine A2 receptors [43]. Under pathological pain states, ROS has been involved in the activation of TRP channels [44, 45], enhancement of NMDA receptor phosphorylation [46], induction of AMPA receptor trafficking to the membrane [47], and activation of MAP kinases [48]; delivery of antioxides could reverse the above changes. However, which downstream mechanisms could underlie the analgesic effect of SANR in low back pain needs to be further studied in the future.

In conclusion, the present study firstly synthesized a novel compound SANR by introducing functional moiety of NSAIDs salicylic acid into NIT nitroxides and demonstrated the dramatic analgesic effects of this newly synthesized compound in low back pain. It was further revealed that this analgesia is at least in partial mediated by a reduction of ectopic spontaneous discharges by SANR in injured DRG neurons. Therefore, synthesis of new NIT nitroxide with NSAIDs salicylic acid framework may represent a novel potential therapeutic candidate for the treatment of chronic pain, including radicular low back pain.
